# Bacteriological qualities and antibiogram studies of bacteria from "suya" and smoked fish (*Clarias gariepinus*) in Dutsin-Ma, Katsina State, Nigeria

**DOI:** 10.11604/pamj.2019.33.219.17729

**Published:** 2019-07-17

**Authors:** Ayodele Timilehin Adesoji, Jude Prince Onuh, Aisha Omokhefue Musa, Peter Femi Akinrosoye

**Affiliations:** 1 Department of Microbiology, Federal University Dutsin-Ma, Dutsin-Ma, Katsina State, Nigeria; 2 Department of Microbiology, University of Ibadan, Ibadan, Oyo State, Nigeria

**Keywords:** Multidrug resistant, suya, smoked fish, antibiogram

## Abstract

**Introduction:**

"suya" and smoked fish are cherished food delicacies in Nigeria, but can be a source of dissemination of Multi-drug Resistant (MDR) bacteria. Moreover, there are limited studies on these MDR bacteria from Dutsin-Ma. Therefore, this study examined the bacteriological qualities and antibiogram profiles of bacteria in these foods from this area in Nigeria.

**Methods:**

Twenty samples of each of "suya" and smoked fish were collected from the study areas and microbiologically analyzed. Total viable count, coliform count, characterization and identification of bacteria were carried out by standard microbiological techniques.

**Results:**

Findings revealed that "suya" samples possessed the highest total viable bacteria count (3.4×10^5^ to 7.7×10^5^ cfu/g) and coliform count (2.1×10^5^ to 6.2×10^5^ cfu/g). A total of 85 and 78 bacteria were isolated from "suya" and smoked fish samples respectively. *E. coli* (24.7% and 24.4%) was the most frequently isolated from each sample respectively. Highest (66.7%) resistance to each of cefuroxime, gentamicin, amoxillin/clavulanate and ciprofloxacin were observed among *E. coli* from "suya". MDR phenotypes commonly isolated was resistance to ceftazidime, cefuroxime, ampicillin, ciprofloxacin, augmentin and nitrofurantoin.

**Conclusion:**

These studies showed the presence of MDR bacteria in samples, hence, raise the need for improved production hygiene and public health awareness.

## Introduction

Animals are a great source of protein. When eaten as food (especially fish), they can make up over 60% of the dietary protein intake by adults, especially in rural areas [1]. In Nigeria, "suya" and smoked fish form a much-cherished delicacy that cuts across socio-economic, age, religious and educational barriers [2-4]. "suya" meat has been described by [4] as a boneless lean meat of mutton, beef, goat or chicken meat stacked on sticks, coated with sauces, oiled and then roasted over wood using a fire from charcoal. It got its name from the Hausa people of Northern Nigeria, and is usually prepared spiced, barbecued, smoked or roasted.

On the other hand, fish smoking is traditionally performed in kilns of clay, cement blocks, drums or iron sheets over a fire to eliminate its moisture content, allowing the product to be stored over a long period of time in market stalls [5]. Moreover, often times these storage facilities are poorly built and can introduce contamination [6]. The presence of *Staphylococcus* spp, *Salmonella* spp, *Streptococcus* spp, *Enterobacter spp, Proteus* spp, *Bacillus* spp. *Pseudomonas* spp, and even fecal *E. coli* have been reported from both "suya" and smoked fish. Many of which have demonstrated multidrug resistance to tested antibiotics [7, 8]. The public health implication of this cannot be overemphasized, with regards to the transferability of the resistant genes.

Animals are known to constitute a vast reservoir of drug resistant enteric bacteria [9-12], and infections/diseases that arise from the consumption of these MDR bacteria-laden animals can lead to failure of conventional treatments, longer treatments and death. Even worse still, they may serve as a potential transfer route of the antibiotic resistant bacteria and resistant genes into human food-chain and environment. Hence, considering their fast emergence in recent times, causing both community-acquired and nosocomial infections [13, 14], and the paucity of information about their activity in "suya" and smoked fish with respect to Dutsin-Ma Local Government Area of Katsina State, it therefore becomes imperative to investigate and report this for public health enlightenment. Therefore, this paper aims at examining the bacterial status and antibiogram profiles of MDR bacteria from "suya" meats as well as smoked fishes from Dutsin-Ma Local Government Area of Katsina State, Nigeria.

## Methods

### Study area and description

This study was conducted in Dutsin-Ma, Dutsin-Ma is a Local Government Area (LGA) in Katsina State, Nigeria. It is located on latitude and longitude 12°27'18''N, 7°29'29''E respectively. The LGA has an area of 527 km^2^ and population of 169,671 as of 2006 census, with Zobe Dam lying to the south of the town [15]. The inhabitants of the local government are predominantly Hausa and Fulani by tribe. Their main occupation is farming and animal rearing.

### Sample collection

In this study, 5 of each of "suya" and smoked fish (*Clarias gariepinus*) samples were randomly collected per week for 4 weeks making a total of 40 samples (i.e. 20 "suya" samples and 20 smoked fish samples). "suya" samples were collected from 5 locations that include: Wednesday market, hospital road, Gawo road, Hanyen Gada and Dan Rimi while smoked fish samples were randomly collected from five other outlets within the study area. All samples were collected between July and August 2017. After collection, samples were immediately wrapped in sterile aluminum foil paper to prevent contamination and transported immediately to the Laboratory of Department of Microbiology, Federal University Dutsin-Ma, Katsina State, Nigeria for microbiological analysis.

### Determination of total viable count

Small pieces of each sample were mashed in a sterile laboratory type mortar with pestle. A gram of the mashed samples was weighed aseptically and then aseptically introduced into 9ml of sterile distilled water, properly shaken before a five-fold dilution was carried out in different test tubes. One milliliter of each of dilution factor 10^1^ and 10^3^ was pipetted and plated out on nutrient agar and MacConkey agar respectively, using spread plate method. Incubation at 37°C for 24 hours was thereafter carried out. Visible colonies were counted to obtain total viable count on each agar plate for determination of total viable count and total coliform count. Discrete colonies were picked out after observing morphologically then, purified by re-streaking on nutrient agar plates before storing on nutrient agar slants at 4°C for further biochemical characterization and identification [16].

### Bacteria characterization and identification

Test organisms isolated were subjected to various biochemical tests such as: gram-stain, motility test, urease test, indole test, methyl-red, vogues proskauer test, citrate test, oxidative fermentation test, triple sugar iron agar test for biochemical characterization, and identified according to the method of Buchanan and Gibbons [16].

### Determination of antibiotic-resistant profiles of isolates

Antibiotic resistant profiles of bacteria were determined by disc diffusion method with antibiotic sensitivity disc (Abtek Biological, Ltd). Two sets of disc were used in this study i.e. gram positive and gram negative bacteria sensitivity discs. Antibiotics present on gram negative disc with their various concentrations include: ceftazidime (30μg), cefuroxime (30μg), gentamicin (10μg), ciprofloxacin (5μg), oflaxacin (5μg), amoxicillin clavulanate (30μg), nitrofurantoin (300μg) and ampicillin (10μg) while that contained on gram positive disc include: erythromycin (5μg), cloxacillin (5μg), Augmentin (30μg), ceftriaxone (30μg) ceftazidime (30μg), cefuroxime (30μg), gentamicin (10μg) and oflaxacin (5μg). In the procedure, stock bacteria cultures that had been purified and stored on nutrient agar slant at 4°C, were inoculated into different 9ml of sterile nutrient broths in separate test tubes and thereafter incubated at 37°C in an incubator overnight. Afterwards, 1ml of a fold dilution was inoculated into sterile distilled water and transferred into different sterile petri dishes each. Sterile Mueller-Hinton agar that has been previously prepared and cooled down to 55°C in water bath was poured into each petri dish and allowed to solidify. However, another 1 in 10 dilution factor was also plated out on nutrient agar for each test for the determination of viable count so as to know the average number of colony used for the susceptibility tests. Antibiotic sensitivity discs were later aseptically placed on each solidified Mueller-Hinton agar plate and incubated at 37°C in an incubator overnight. Diameter of zones of inhibition seen round the antibiotics sensitivity discs were measured with a meter rule and categorized as resistant, intermediate and sensitive based on the Clinical and Laboratory Standards Institute (CLSI) standard for each bacteria isolate [17]. However, only the resistant organisms were recorded in this study.

## Results

The study reveals that among the "suya" samples collected, samples from Wednesday market possessed the highest total viable bacteria count (3.4×10^5^ to 7.7×10^5^ cfu/g) and coliform count (2.1×10^5^ to 6.2×10^5^ cfu/g), while the least total viable count (1.6×10^5^ to 2.8×10^5^ cfu/g) and coliform count (1.7×10^5^ to 3.7×10^5^ cfu/g) were observed from samples collected along the hospital road. Whereas, in the smoked fished samples, the highest (4.8 x10^5^ to 6.5x10^5^ cfu/g) total viable count was observed among samples collected from outlet D, and the least (4.8 x10^5^ to 6.5 x10^5^ cfu/g) from samples collected from outlet A (1.50 x10^5^ to 2.9 x10^5^ cfu/g). The highest (4.0 x10^5^ to 7.6 x10^5^ cfu/g) and lowest (1.6 x10^5^ to 2.3 x10^5^ cfu/g) coliform count was however observed from samples collected from outlet E and C respectively (Table 1).

**Table 1:** Range of bacterial count (cfu/g) from "suya" and smoked fish from different sources in Dutsin-Ma

Samples	Sources	Total viable counts	Coliform counts
**Suya**	Hospital Road	1.6×10^5^ - 2.8×10^5^	1.7×10^5^ - 3.7×10^5^
	Gawo Road	1.4×10^5^ - 3.4×10^5^	1.3×10^5^ - 3.8×10^5^
	Hanyen Gada	1.0×10^5^ - 5.0×10^5^	2.9×10^5^ - 4.1×10^5^
	Wednesday market	3.4 ×10^5^ - 7.7 ×10^5^	2.1×10^5^ - 6.2×10^5^
	Dan Rimi	4.2 ×10^5^ - 7.2 ×10^5^	1.2×10^5^ - 5.3×10^5^
**Smoked fish**	Outlet A	1.5 x10^5^ - 2.9 x10^5^	1.4 x10^5^ - 2.7 x10^5^
	Outlet B	3.4 x10^5^ - 6.2 x10^5^	2.0 x10^5^ - 3.2 x10^5^
	Outlet C	1.6 x10^5^ - 3.3 x 10^5^	1.6 x10^5^ - 2.3 x10^5^
	Outlet D	4.8 x10^5^ - 6.5 x10^5^	3.5 x10^5^ - 6.4 x10^5^
	Outlet E	2.2 x10^5^ - 3.6 x10^5^	4.0 x10^5^ - 7.6 x10^5^

**Figure 1:**
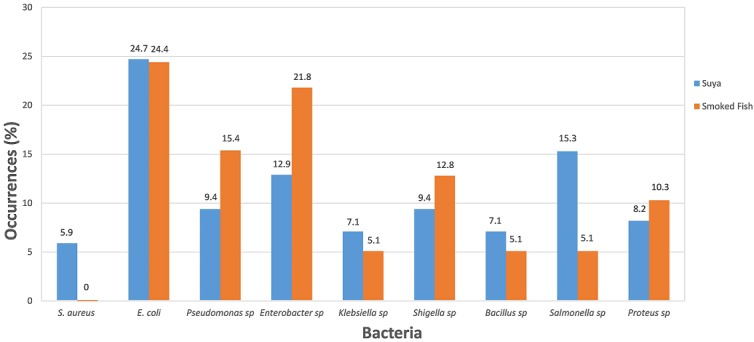
Percentage occurrence of bacteria isolated from "suya" and smoked fish

A total of 163 bacteria were isolated from this study. This include *E. coli* (24.5%), *Pseudomonas* spp (12.3%), *Klebsiella* spp (6.1%), *Proteus* spp (9.2%), *Shigella* spp (11.0%), *Salmonella* spp (10.4%), *Bacillus* spp (6.1%) and *Enterobacter* spp (17.2%). *E. coli* was the most prevalent bacteria isolated from the samples, with the highest (24.7% and 24.4%) occurrences in the "suya" and smoked fish samples respectively (Table 2, Figure 1). While *S. aureus* had the least (5.9%) prevalence in the "suya" samples, the least (5.1%) isolated organisms from the smoked fish samples were *Klebsiella* spp, *Salmonella* spp and *Bacillus* spp. (Table 2). The results of the antibiotic resistance profiling of isolated bacteria revealed that most of the isolated bacteria demonstrated resistance to ceftazidime, cefuroxime, gentamicin, ciprofloxacin, amoxicillin clavulanate, ampicillin and nitrofurantoin (Table 3). Judging from their resistance to three or more classes of antibiotics, multidrug resistance was observed among *Enterobacter* spp, *Proteus* spp, *E. coli*, *Shigella* spp and *Bacillus* spp, isolated across all the "suya" and smoked fish sampled, except for *Klebsiella* spp and *Pseudomonas* spp, which were resistant only to about two classes of antibiotics. The resistant phenotypes of these organisms reveal that the most prevalent multidrug phenotype is found among *E. coli* with a 37.1% occurrence in smoked fish and 21.7% in "suya" samples (Table 4).

**Table 2:** Bacteria isolates from "suya" meats and smoked fish from different sources in Dutsin-Ma

	Sources (“Suya”)							Outlets (Smoked fish)						
Bacteria	Hosp. Road	Gawo road	Hayen Gada	Wed. Market	Dan Rimi	Total	%	A	B	C	D	E	Total	%
*Staphylococcus sp*	1	ND	1	2	1	5	5.9	-	-	-	-	-	-	-
*E. coli*	3	4	2	8	4	21	24.7	5	2	5	4	3	19	24.4
*Pseudomonas sp*	2	3	ND	ND	3	8	9.4	3	2	2	2	3	12	15.4
*Enterobacter sp*	5	2	1	3	ND	11	12.9	4	4	2	5	2	17	21.8
*Klebsiella sp*	1	ND	2	3	ND	6	7.1	2	ND	ND	2	ND	4	5.1
*Shigella sp*	2	3	ND	2	1	8	9.4	2	5	ND	2	1	10	12.8
*Bacillus sp*	ND	2	1	3	ND	6	7.1	ND	2	ND	1	1	4	5.1
*Salmonella sp*	1	3	1	5	3	13	15.3	2	ND	ND	2	ND	4	5.1
*Proteus sp*	2	2	ND	1	2	7	8.2	ND	2	1	2	3	8	10.3
**TOTAL**						**85**	**100**						**78**	**100**

**Key** ND= Not detected

**Table 3:** Antibiotic-resistant profiles of isolated bacteria from "suya" and smoked fish from various sources in Dutsin-Ma

				NUMBER (%) RESISTANT TO:								
SAMPLE	BACTERIA	CAZ	CRX	GEN	OFL	AUG	CPR	NIT	AMP	CXC	ERY	CTR
**Suya**	*E.coli*	7 (33.3)	14 (66.7)	14 (66.7)	4 (19.0)	14 (66.7)	14 (66.7)	10(47.6)	12 (57.1)	-	-	-
	*Pseudomonas spp*	5 (63)	6 (75)	2 (25)	8 (100)	0	8 (100)	0	6 (75)	-	-	-
	*Enterbacter spp*	8 (72.7)	9 (82)	6 (55)	3 (27.3)	5 (45.4)	7 (64)	2 (18)	4 (36)	-	-	-
	*Klebsiella spp*	6 (100)	0	5 (83.3)	1 (16.7)	6 (100)	1 (16.7)	5 (83.3)	3 (50)	-	-	-
	*Shigella spp*	6 (75)	8 (100)	6 (75)	3 (37.5)	4 (50)	4 (50)	6 (75)	4 (50)	-	-	-
	*Salmonella spp*	8 (61.5)	7 (54.8)	9 (69.2)	6 (46.1)	4 (30.8)	4 (30.8)	0	0	-	-	-
	*Proteus spp*	6 (86)	4 (57)	5 (71)	5 (71)	0	1 (14)	5 (71)	6 (86)	-	-	-
***Smoked fish***	*Klebsiella spp*	4 (100)	2 (50)	0 (0)	1 (25)	2 (50)	4 (100)	2 (50)	4 (100)	-	-	-
	*Pseudomonas spp*	3 (25)	6 (50)	3 (25)	3 (25)	9 (75)	0 (0)	6 (50)	9 (75)	-	-	-
	*Enterobacter spp*	5 (29.4)	7 (42.2)	6 (35.3)	8 (47.1)	5 (29.4)	5 (29.4)	6 (35.3)	10 (58.8)	-	-	-
	*Proteus spp*	8 (100)	2 (25)	4 (50)	6 (75)	2 (25)	5 (67.5)	5 (67.5)	4 (50)	-	-	-
	*Salmonella spp*	0 (0)	4 (100)	2 (50)	4 (100)	1 (25)	2 (50)	0 (0)	0 (0)	-	-	-
	*E. coli*	6 (31.5)	12(63.2)	12(63.2)	4 (21.0)	12(63.2)	12(63.2)	9 (47.4)	11 (57.8)	-	-	-
	*Shigella spp*	8 (80)	10 (100)	8 (80)	7 (70)	4 (40)	5 (50)	3 (30)	6 (60)	-	-	-
	*Bacillus*	1 (25)	0 (0)	2 (50)	1 (25)	0 (0)	-	-	-	0 (0)	0 (0)	4 (100)

**KEYS: CAZ=** Ceftazidime (30μg)**, CRX=** Cefuroxime (30μg)**, GEN=** Gentamicin (10μg)**, CPR=** Ciprofloxacin (5μg)**, OFL=** Ofloxacin (5μg)**, AUG=** Amoxillin Clavulanate (30μg)**, NIT=** Nitrofurantoin (300μg)**, AMP=** Ampicillin (10μg)**, ERY=** Erythromycin (5μg), **CXC =** Cloxacillin (5μg), **CTR**=Ceftriaxone

**Table 4:** Phenotypes of Multidrug resistant bacteria from smoked fish and "suya" from Dutsin-Ma metropolis

	Smoked Fish		Suya	
BACTERIA	No. (% MDR)	RESISTANT PHENOTYPES	No. (% MDR)	RESISTANT PHENOTYPES
*Enterobacter*spp	11 (31.4)	CRX, AMP, OFL	7 (15.2)	CRX, CPR, AUG, GEN
*Proteus* spp	2 (5.7)	CAZ, CPR, AMP, OFL, NIT, GEN	4 (8.7)	CAZ, CRX, AMP, NIT, GEN
*E. coli* spp	13 (37.1)	CRX,CPR,AMP,NIT,GEN,AUG,	10 (21.7)	CAZ, CPR, AMP, NIT, GEN, AUG
*Shigella* spp	1 (2.9)	CAZ,CRX,CPR,AMP,OFL,GEN	5 (10.9)	CAZ, CRX, CPR, NIT, GEN, AUG
*Bacillus* spp	ND	ND	3 (6.5)	CAZ, OFL, NIT, AUG, ERY
*Staphylococcus* spp	ND	ND	3 (6.5)	CAZ, AMP, OFL, NIT, GEN, AUG
**TOTAL**	**35 (100)**		**46 (100)**	

**Keys:** ND= Not detected CAZ= Ceftazidime (30μg), CRX= Cefuroxime (30μg), Gen = Gentamicine (10μg), CPR = Ciprofloxacin (5μg), OFL= Ofloxacin (5μg), AUG= Amoxycillin clavulanate (30μg), NIT= Nitrofurantoin (300μg), AMP= Ampicillin (10μg), ERY= Erythromycin (5μg), CXC = Cloxacillin (5μg), AUG= Augmentin (30μg), CTR=Ceftriaxone(30μg)

## Discussion

"suya" and smoked fish are increasingly becoming a more and more popular delicacy in Nigeria. Therefore, isolation of bacteria from "suya" and smoked fish should raise public health concern. Values of total bacteria count (4.8 x10^5^ to 6.5x10^5^ cfu/g) and total coliform count (4.0 x10^5^ to 7.6x10^5^ cfu/g) obtained from the smoked fish samples from this study have been identified to be higher than standard microbiological load (≥10^4^) of ready-to-eat food [18]. The "suya" meat samples from the Wednesday market had the highest range of bacteria load in cfu/g. Its total viable count ranged from 3.4×10^5^ to 7.7×10^5^ cfu/g while coliform counts ranged from 2.1×10^5^ to 6.2×10^5^ cfu/g (Table 1). This agrees with the findings of Amadi *et al*. [19], who also reported a high range in coliform count (1.5×10^4^ - 6.2×10^4^ cfu/g) in a study conducted in Rivers State, Nigeria. The finding reports the presence of *S. aureus, E. coli, Pseudomonas* spp, *Enterobacter* spp, *Klebsiella* spp, *Shigella* spp, *Bacillus* spp, *Salmonella* spp and *Proteus* spp in the "suya" and smoked fishes sampled (Table 2). This is consistent with the report of Egbebi and Muhammed [20], who isolated *Staphylococcus* spp, *E. coli, Pseudomonas* spp from ready-to-eat "suya" meat sold in Owo, Ondo State, Nigeria and that of Chukwura and Mojekwu [21], who also stated that microbiological analysis of "suya" meat samples in Enugu State, Nigeria showed a contamination of meat samples with various bacterial species including *S. aureus* and some enteric bacteria. Adams and Moss [7] and Abdullahi *et al.* [22] also stated that the presence of *Salmonella* spp as contaminant could be attributed to inadequate heating of meat product during its preparation.

Contrary to the report of Egbebi and Muhammed [20], who reported least (15%) occurrence of *E. coli*, our findings revealed that *E. coli* was the most prevalent bacteria isolated from the samples, with the highest occurrence in the "suya" (24.7%) and smoked fish (24.4%) samples isolated across all the markets and outlets sampled (Figure 1). This phenomenon suggests fecal contamination of sample. While *S. aureus* had the least (5.9%) prevalence in the "suya" samples, the least (5.1%) isolated organisms from the smoked fish samples were *Klebsiella* spp, *Salmonella* spp and *Bacillus* spp. (Table 2). This is also contradicting the report of Nwakanma *et al*. [23] and Egbebi and Muhammed [20], who attributed the highest (35%) prevalence of *Staphylococcus* spp to contamination from the handlers. However, the high bacteria count observed among the "suya" and smoked fish samples from our study may also be attributed to the poor hygienic condition under which they are produced (as observed during the time of sample collection) i.e. open space where they were sold and stored [24, 25]. It is also imperative to note that the observed high microbial counts may be due to the original bio-load of slaughtered sick animals, the transportation by rickety vehicle and use of contaminated equipment [4]. These reported values, therefore, place the "suya" and smoked fish samples examined in this work in the "acceptable but not satisfactory" range (10^5^-10^7^ for "suya" and 10^6^-10^7^ for smoked fish) under the Public Health Laboratory Service guidelines for the bacteriological quality of ready-to-eat foods samples at the point of sale [18].

Whereas the presence of some members of the family of *Enterobacteriaceae* may be due to contamination from long exposure of the "suya" meat to air, the organisms isolated in this study are the organisms usually suspected to be in connection with meat contamination and spoilage [26]. When these findings were compared to that from smoked fish samples, an almost similar result was observed. *E. coli* was the highest isolated organism from "suya" (24.4%) and smoked fish (24.7%) samples, whereas *Klebsiella* spp and *Bacillus* spp demonstrated least occurrence in both "suya" (5.1%) and smoked fish (7.1%) samples. The study revealed a 5.7% occurrence of *Staphylococcus* species, a normal bacteria flora on human skin [27] on the "suya" samples, and no isolation of the organism from smoked fish. This could be due to cross contamination of the "suya" samples, from vendors' hand during handling, especially after long preservation time. Gilbert and Harrison [27] also stated that "suya" meat preserved with a certain amount of salt may also permit the growth of *S. aureus*. Most of the bacteria (*S. aureus*, *E. coli*, *Enterobacter* spp, *Shigella* spp, *Bacillus* spp, *Salmonella* spp and *Proteus* spp) isolated demonstrated multidrug resistance except *Klebsiella* spp and *Pseudomonas* spp. The prevalence of multidrug resistant *E. coli* (37.1% prevalence in smoked fish and 21.7% in "suya" samples) and *Enterobacter* spp (31.4% prevalence in smoked fish and 15.2% in "suya" samples) reported from this study, agrees with a similar observation reported by Eze *et al.* [28]. In an assessment of "suya", frozen and dried fish, for multidrug resistant bacteria, conducted in Obollor-afor and Nsukka, Enugu State, Nigeria, Eze *et al*. reported that strains of *E. coli* demonstrated more than 36% resistance to 7 antibacterial agents tested. *Salmonella* isolates also exhibited more than 33% resistance to 9 antibacterial agents. However, contrary to our findings, they reported that more than 60% of the *Pseudomonas* isolates were resistant to 8 antibiotics tested.

The public health implication of this is of great concern. This is even more glaring in the light of the transferability of these resistance traits among both pathogenic and potentially pathogenic bacteria [28]. In a study conducted by Egbebi and Seidu [3] on "suya" sold in Ado and Akure, Southwest Nigeria, bacteria, as well as molds, yeast and fungi have been reported.

## Conclusion

Despite the wide spread popularity of "suya" and smoked fish delicacies in Nigeria, MDR bacteria with the ability to endanger human lives have been reported in high numbers from the samples studied. Hence, a great public health concern which calls for antibiotic resistant bacteria surveillance among clinicians and public health practitioners in this vicinity. While proper hygiene of the vendors, the processing environment and process-line of suya is highly recommended to be clean, the practice of preparation and distribution of "suya" and smoked fish in open places where there is no emphasis on hygiene standards should be discouraged. Proper sensitization of the local vendors on proper animal husbanding, hygienic slaughter and storage of meat, sanitation of utensils and equipment would help decrease the rate of infections from these foods. While a reduction in exposure to antibiotics is greatly advised, development of new and more effective antibiotics is recommended.

### What is known about this topic

- "suya" and smoked fish are a widely consumed delicacy in Nigeria;
- They can be sources of pathogenic, drug resistant enteric bacteria.

### What this study adds

- Bacteriological qualities and antibiotic resistant profiles of "suya" and smoked fish in Dutsin-Ma Local Government Area of Katsina State, Nigeria were determined.

## Competing interests

The authors declare no competing interests.
